# Botulinum Toxin for the Treatment of Hemifacial Spasm: An Update on Clinical Studies

**DOI:** 10.3390/toxins13120881

**Published:** 2021-12-09

**Authors:** Nicola Tambasco, Marta Filidei, Pasquale Nigro, Lucilla Parnetti, Simone Simoni

**Affiliations:** 1 Movement Disorders Center, Neurology Department, Perugia General Hospital and University of Perugia, 06129 Perugia, Italy; pasquale.nigro1987@gmail.com; 2 Neurology Department, Perugia General Hospital and University of Perugia, 06129 Perugia, Italy; martafilidei91@gmail.com (M.F.); lucilla.parnetti@unipg.it (L.P.); simonesimoni84@gmail.com (S.S.)

**Keywords:** hemifacial spasm, botulinum toxin, BoNT, spasm, onabotulinum toxin, abobotulinum toxin

## Abstract

Hemifacial spasm (HFS) is a movement disorder characterized by involuntary contractions of the facial muscles innervated by the seventh cranial nerve. Generally, it is associated with a poor quality of life due to social embarrassment and can lead to functional blindness. Moreover, it is a chronic condition, and spontaneous recovery is rare. Intramuscular injections of Botulinum Toxin (BoNT) are routinely used as HFS treatment. Methods: We reviewed published articles between 1991 and 2021 regarding the effectiveness and safety of BoNT in HFS as well as any reported differences among BoNT formulations. Results: The efficacy of BoNT for HFS treatment ranged from 73% to 98.4%. The mean duration of the effect was around 12 weeks. Effectiveness did not decrease over time. Adverse effects were usually mild and transient. The efficacy and tolerability of the different preparations appeared to be similar. Among the studies, dosage, injected muscles, intervals of treatment, and rating scales were variable, thus leading to challenges in comparing the results. Conclusions: BoNT was the treatment of choice for HFS due to its efficacy and safety profile. Further studies are needed to investigate the factors that influence the outcome, including the optimal timing of treatment, injection techniques, dosage, and the best selection criteria for formulations.

## 1. History Presentation and Epidemiology

Hemifacial spasm (HFS) is a hyperkinetic movement disorder characterized by short or persistent, intermittent synchronous twitching of the muscles innervated by the facial nerve [1], which is a chronic condition, and spontaneous recovery is rare [2,3]. Typical features include involuntary clonic and/or tonic contractions of the muscles of facial expression, usually unilaterally, initiating in the periorbital musculature, progressing to involve the perioral, platysma, and other muscles of facial expression [1,4]. HFS interferes with social life in about 90% of patients, leading to isolation and even depression, there, in turn, having a negative impact on the quality of life [5]. Therefore, early diagnosis and optimal therapy are generally necessary.

HFS was described for the first time by F. Schultze, in 1875, in a 56-year-old male having involuntary movements involving the left side of his face with post-mortem examination of a giant aneurysm of the left vertebral artery compressing the left facial nerve [6]. The condition received its current terminology by Babinski in 1905 [7].

Currently, HFS is classified as primary (79%) or secondary to facial nerve damage (21%) [8]. The former is attributed to the compression of the facial nerve at the root exit zone in the brainstem, usually by an ectatic or aberrant blood vessel [8,9]. Instead, the latter has been associated with a number of conditions, including cerebellopontine angle tumors, acoustic neuroma or meningioma, epidermoid, arachnoid cyst, lipoma, arteriovenous malformations; brainstem lesions (stroke, trauma, demyelinating disorders, tumors), infections (otitis media, tubercular meningitis), structural abnormalities of the posterior cranial fossa (Paget’s disease, Chiari malformation); parotid tumors; and Bell’s palsy [10]. 

The mean prevalence of the disorder is around 10 in 100,000 (14.5 and 7.4 per 100,000 in females and males, respectively) [11,12]. The average age at onset of primary HFS ranges from the fifth to sixth decades of life [8]. HFS is commonly sporadic, with a few familial cases having been reported [13,14]. Likewise, the bilateral disease is also rare (2.6%), and when it does present it, begins unilaterally, progressing to involve the other side [15].

Diagnosis of HFS is mainly based on clinical recognition. Additionally, detailed patient history and a neurological examination are required to exclude any signs which might suggest an underlying secondary cause. Currently, available diagnostic work-up includes electromyography to exclude denervation due to facial nerve lesions, as well as brain MRI to rule out any demyelination or space-occupying lesions near the brainstem [16]. The distinction between primary or secondary HFS is fundamental to properly direct the treatment strategy. Indeed, whereas the management of primary HFS reduces the patient’s symptoms and eventually can lead to consistently resolute the clinical picture, in secondary HFS, the focus should be first at identifying and treating the underlying cause. Differential diagnosis of HFS needs to be made for blepharospasm, tardive dyskinesias, motor tics, psychogenic HFS, focal cortical seizures involving the facial muscles, and aberrant regeneration after facial nerve injury.

Among the available clinical scales, the Hemifacial Spasm Grading Scale (HSGS) is regarded as an objective, quick and reliable tool for the assessment of HFS, based upon motor signs which are useful for monitoring Botulinum toxin (BoNT) treatment efficacy over time [17]. Differently, the seven-item HFS-7 measures the quality of life, but none of the motor skills [18]. Moreover, the HFS Score has been developed by incorporating both motor and subjective features [19].

Several drugs have been studied for the treatment of HFS, such as anticonvulsants including carbamazepine, clonazepam, gabapentin, and others including baclofen, anticholinergics, and haloperidol [1]. Reliable data on the efficacy of oral treatment has been limited, and numerous side effects have been reported, such as sedation and fatigue [20].

Concerning treatment, the most efficacious therapy has been reported to be BoNT, a biological toxin derived from the Clostridium botulinum [2,21]. It is active on the presynaptic region of the neuromuscular junction, inhibiting the calcium-mediated release of acetylcholine at the nerve terminal, therein avoiding impulse generation downstream, resulting in neuroparalysis [22].

The regulation of a fusion of the synaptic vesicle with the plasma membrane involves a complex group of proteins referred to as SNAREs (soluble N-ethylmaleimide sensitive factor attachment protein receptor) [23,24].

The active form of BoNT is a di-chain polypeptide, composed of two chains: a heavy chain (HC) (100 kDa) and a light chain (LC) (50 kDa), associated with auxiliary proteins including haemagglutinins and non-haemagglutinins [25,26]. The LC, which is a zinc protease, performs the key action of the toxin by cleaving SNARE proteins [27]. The carboxy-terminal end of the HC domain binds to a polysialoganglioside receptor on the presynaptic membrane. Likewise, BoNT-A binds to the synaptic vesicle (SV2) protein receptor and BoNT-B to synaptotagmin, both located either inside the exocytosed synaptic vesicle or on the presynaptic membrane. The BoNT is endocytosed inside synaptic vesicles. The LC, in the cytosol, is released from the HC and cleaves the interchain disulphide bond of the SNARE protein, thus preventing neurotransmitter release and therein leading to neuroparalysis [22].

There are seven immunologically distinct BoNT serotypes (labelled A to G) that cleave specific SNARE proteins. To date, there are only two commercially available BoNT serotypes, botulin toxin type A (BoNT-A) and BoNT type B (BoNT-B).

Despite limited data from high-quality clinical trials, BoNT-A is considered the treatment of choice for HFS patients [28,29]. Overall, 76% to 100% of patients have at least a 75% improvement with a typical duration of response lasting from 3 to 4 months [30,31]. Both primary and secondary HFS patients respond to BoNT-A [10]. Average efficacy has been reported to be around twenty years, often with the need for a gradual increase in dose [4,30,32].

Surgery represents an alternative option for the treatment of HFS. Surgical treatment is a definitive therapy, whereas BoNT provides only a temporary remedy. The first line surgical procedure is microvascular decompression (MVD) of the facial nerve, which consists of removing the compression of the seventh nerve at the root exit zone by the aberrant/ectatic vessel. MVD for HFS was suggested for the first time by Gardner in 1962 [33] and then described further by Jannetta et al. [34].

A standard retrosigmoid craniectomy or craniotomy is used to expose the facial and vestibulocochlear nerves, as well as the lower cranial nerves. The aberrant vessel is identified and dissected away from the facial nerve; shredded Teflon or a Teflon patty is placed between the artery and nerve to assure adequate decompression. Intraoperative neuromonitoring with motor evoked potentials and brainstem auditory evoked potentials is used for making sure that the cranial nerves are functioning and that an adequate de-compression has been obtained. The resolution of an abnormal muscle response on electromyography following decompression confirms the absence of a zone of vascular compression. A recent meta-analysis reported that the overall spasm freedom rate after MVD was 90.5% at the last follow-up of 1.25 ± 0.04 years [35]. The most frequent complications are transient or permanent cranial nerve deficits, leading to hearing loss or facial weakness and CSF leak [35]. The surgical treatment can be reserved for those patients who do not respond to BoNT therapy or desire a long-lasting solution.

## 2. Reported Trials—Evidence-Based Medicine

This review focused on the efficacy and safety of BONT for the treatment of HFS from studies by collecting data from studies published between 1991 to 2021 (Table 1).

From the literature, the efficacy of BONT ranged from 73% to 98.4%. The mean duration of the effect was around 12 weeks. There were three RCT studies, of which two were conducted to investigate the efficacy and safety of BoNT in HFS [36,37]. The third study compared pretarsal versus preseptal injections of the orbicularis oculi in 31 patients with HFS reporting that the pretarsal portion of the orbicularis oculi was associated with a significantly high response rate in terms of latency to response, duration of improvement, Jankovic Rating Scale (JRS), self-response scale, and patient satisfaction scale than the preseptal injections [38]. The remaining studies were not randomized or prospective. A retrospective longitudinal comparative analysis suggested that the duration of relief from symptoms remains unchanged over the long term in patients with HFS [39]. Moreover, Tunc et al. assessed BoNT injections efficacy in 69 patients with primary HFS (*n* = 46) and those with HFS due to definite neurovascular compression (*n* = 23), reported that primary HFS patients presented more improvement [40] (Table 1).

The second type of study was carried out to compare effectiveness and safety among different formulations of BoNT (Table 2).

Of these 12 studies, all investigated OnabotulinumtoxinA. Seven experimental studies explored the clinical differences between OnabotulinumtoxinA vs. AbobotulinumtoxinA. Among these, only one study reported that Ona was superior to Abo in a prospective, single-blind, multicenter study. The only RCT study comparing Ona and Abo did not observe any differences. Among the five remaining, three studies investigated OnabotulinumtoxinA and LanbotulinumtoxinA and two OnabotulinumtoxinA and IncobotulinumtoxinA (Table 2). Inco seemed to have a slightly higher subjective improvement and longer duration of the effect compared to Ona. However, the study included a small number (=12) of patients [62]. A prospective, randomized, double-blind study comparing Lan and Ona reported that these formulations had similar efficacies (mean duration effect: 12.8 vs. 12.9, respectively), no significant difference in safety, along with similar tolerability profiles (excellent for 25.5% Ona and 5.3% Lan, good for 64.7% Ona and 57.9% Lan), so that a dose equivalence of 1:1 may be considered for HFS treatment [60]. Additionally, Wu et al. compared the therapeutic efficacy and safety profiles of Lan (CBONT-A, Lanzhou Biological Products Institute, China) and Ona for the treatment of HFS, reporting no significant differences in the two rates [61].

## 3. Treatment Challenges and Pitfalls

Botulinum therapy has some limitations. Most importantly, the response rate is high, about 97% [2]. Moreover, it requires repeated administrations at three- and six-months intervals. Even though the safety profile is considered favorable [30,64], adverse effects have been reported, including mild facial paresis (23%), diplopia (17%), and ptosis (15%) [1]. Additionally, trauma from subcutaneous injections can provoke transitory bruising. Regarding systemic adverse effects, flu-like symptoms have been reported in about 14% of patients within 24 h after BoNT injection (1.7–20% of patients treated with BoNT-A and in 5–55% of those treated with BoNT-B) [43]. The immunoresistance to VTx is unlikely in cases of HFS due to a low dosage employed. The main long-term side effect is facial asymmetry, which can be solved with an injection of the not affected side [37]

Moreover, treatment pitfalls regarding the efficacy and safety of BoNT have known challenges requiring the delivery of tailored dosages and precise identification of target muscles [4]. Other factors that can affect the efficacy of BoNT include volume and dilution, convection, local temperature, and the response of the treated muscle [65,66]. A 10-year follow-up study reported that BONT effectively induced sustained relief from symptoms of HFS over the long-term, with only minimal and transient adverse reactions [3]. Moreover, HFS patients frequently complain of non-motor and motor-related symptoms that can often be relieved by administering BoNT [11]. Finally, another disadvantage of BONT is its high cost [67].

## 4. Practical Guidelines of Treatment

**Figure 1:**
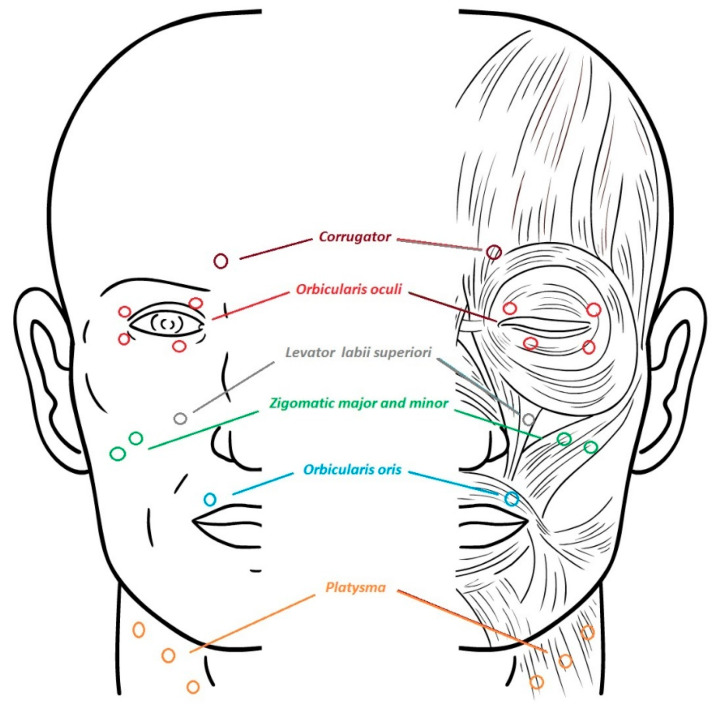
Most frequent treated muscles in hemifacial spasm.

BoNT has to be diluted to a minimal concentration in order to lower its spreading. It is injected via a 30-gauge needle. The muscles involved in HFS include: orbicularis oculi, corrugator supercilii, zygomaticus major, zygomaticus minor, levator labii superioris alaeque nasi, risorius, orbicularis oris, mentalis, depressor anguli oris, and platysma (Figure 1).

In a subgroup of HFS patients, characterized by mild symptomatology, the treatment of orbicularis oculi seemed to be able to control muscle contractions, even in the lower part of the face [4]. This might be due to either the lowering of a triggering spasm from the upper muscles or the spread of the botulinum toxin [48]. In the case of more severe spasms involving the lower facial muscles, a broader treatment targeting the lower facial muscles is recommended [48].

Currently, total doses recommended for HFS for each session should range accordingly: 10–34 U for OnabotulinumtoxinA [68], 53–160 U for AbobotulinumtoxinA [2], and 1250–9000 U for RimabotulinumtoxinB [69]. The therapeutic effect begins at about 3–6 days after treatment and can persist for 2–3 months. Intervals of 3 months between injections are generally recommended. Treatment should be started at low doses and up-titrated, if deemed necessary, depending on the response to therapy.

For HFS, orbicularis oculi injection is similar to that for blepharospasm, although in some patients, injecting the lower portion in the Orbicularis oculi may be sufficient [4]. Specifically, injection of the pretarsal portion may be more effective than preseptal or ocular portion orbicularis oculi injections [38]. Visual inspection is adequate for localizing subcutaneous facial muscles, including orbicularis oculi and platysma. In HFS the problem is to identify from facial expression the muscles and to inject the muscles around the mouth when HFS is severe in the lower territory and keeping at the same time a symmetrical smile. Injection of other facial muscles may first require EMG or electrical stimulation to assure accurate placement.

## 5. Proposal for Research and Future Studies

Presently, international guidelines recommend the use of BoNT for HFS. However, these indications have been based upon observational data. In light of this, randomized trials need to be designed to investigate the factors that influence outcome, including the optimal timing of treatment, injection techniques, dosage and the best selection criteria for formulations. Other aspects that require independent study include patient quality of life, safety, and immunogenicity. The aim of future research should also focus on improving formulations of botulinum toxin, by developing more stable neurotoxins having longer durations and more constant in their efficacy.

## Figures and Tables

**Table 1:** Literature review of published articles regarding Botulinum toxin for HFS.

KERRYPNX	Author	Design	Clinical Evaluation	*n*°	Mean Age	Disease Duration	End-Point	Improvement	BoNT Type	Mean Dosage (UI)	Effect Duration
1	Yoshimura (1992) [36]	P, RCT, DB	10-points RS	11	50	5.2 year	efficacy and safety	79%	Ona	mag-90	2.8 month
2	Berardelli (1993) [41]	P, Mc	Marsden and Schachter-RS	63	n/a	n/a	efficacy	73–81%	Ona	15 to 40 per eye	11 week
3	Park (1993) [42]	P	0–4 point RS	101	53.3	6 month-35 year	efficacy and safety	98.4%	Ona	13.5	16.5 week
4	Jitpimolmard (1998) [2]	P	VAS	158	49.10	4 year	long-term efficacy and safety	97%	Abo	92	3.4 month
5	Thussu (1999) [43]	P	JRS	27	47.78	4.67 year	efficacy	3.78	Abo	74.37	4.46 month
									Ona	12.73	
6	Trosch (2007) [44]	P, OL	VAS	6	60.5	n/a	safety of BoNT-B and dose-finding study	4.3 (100 UI)	Rima	100	n/a
				6				4.7 (200 UI)		200	n/a
				6				4.1 (400 UI)		400	n/a
				6				2.2 (800 UI)		800	n/a
7	Tunc (2008) [40]	P	AIMS	46 (iHFS)	54.4	35.4 month	efficacy (iHFS vs. nHFS)	2.43 (iHFS)	ONA	20	n/a
				20 (nHFS)	50.7	21 month		0.43 (nHFS)			
8	Cillino (2010) [45]	R	n/a	58	71.7	13.3	Long-term efficacy and safety	n/a	Ona	18.7	20.6 week
9	Rudzinska (2010) [11]	P, OL	CGI-S, BDI, NMSQ	56	60	n/a	Efficacy (motor and non- motor symptoms)	75%	BONT-A (Botox-Dysport)	120 (Dysport)	n/a
										25 (Botox)	n/a
10	Bastola (2010) [46]	P	JRS	19	n/a		efficacy	3.9	BONT-A	n/a	5.8 month
11	Gill (2010) [39]	R (early vs. late)	JRS	16	57.6	3.8 year	Long-term efficacy	n/a	BONT-A	32.9 (early sessions)	12.4 week (early)
										38.4 (late sessions)	12.4 week (late)
12	Kollewe (2010) [47]	P	GCI	97	n/a	n/a	Efficacy	2.6	Ona	22	12.1
									Abo	51	12.2
13	Colakoglu (2011) [48]	R, SB, CO	Clinical Grading of Severity in HFS scale	23	61.95	9.26 year	Efficacy into lower facial muscles	1.88 (mild HFS) 2.35 (severe HFS) 2.56 (orb. oculi) 2.17 (perioral)	BONT-A	16.86	15.4 week
14	Ababneh (2014) [30]	R	patients’ satisfaction score	11	73.4	n/a	Dose-finding; efficacy, safety, duration of effects, (first vs. last injections)	3.3 (first year)	Ona	24.9 (first year)	14.1 week (first year)
								3.6 (last year)		28.1 (last year)	18.3 week (last year)
15	Streitova (2014) [32]	R	JRS	18	n/a	>4 year	efficacy and safety	76%	Abo	100–150	12 week
16	Li (2015) [37]	Ra, DB, CO	Cohen grade scale	20	52.9	4.35 year	efficacy and safety	n/a	Lan	47.25 ± 5.5	3–5 month
17	Choe (2016) [49]	P	not specified	23	61.8	n/a	injection strategy	95%	Abo- Ona	28.6	28.6 week
18	Jog (2016) [50]	P, M	SF-6D Health Utility Scores	38 (naive)	62	n/a	QoL	n/a	Ona	n/a	n/a
				78 (maint)	66	72.9 month					
19	Lolekha (2017) [38]	P, Ra, DB, CO	JRS	31	59.77	5.55 year	comparison preseptal vs. pretarsal injection	1.55 JRS (preseptal)	Ona	18.75	9.74 week (preseptal)
								1.23 JRS (pretarsal)			10.32 week (pretarsal)
20	Gutierrez (2021) [51]	R	Chong HFS-RS	162	47.7	12.74 year	Long-term efficacy	78%	Ona	17.9	3.59 month
									Abo	60.9	3.72 month
21	Badarny (2021) [52]	R	Likert scale	42	52	n/a	efficacy	90%	Ona	17.9	n/a
									Abo	60.9	n/a
22	Kongsaengdao (2021) [53]	P	HFS-30, AIMS, SF-36, depression questionnaire	74	60.8	5.27 year	long-term QoL	n/a	Abo	100	3 month
23	Lee (2021) [54]	R	CGI, QoL	184	61.01		Efficacy and long-term adherence	n/a	n/a	n/a	n/a

Abo: AbobotulinumtoxinA; AIMS: abnormal involuntary movement scale; CO: crossover; DB: double-blind; GCI: Global clinical improvement; iHFS: idiopatic hemifacial spasm; JRS: Jankovic Rating Scale; Lan: LanbotulinumtoxinA; Mc: multicenter; nHFS: neurovascular hemiafacial spasm; NMSQ: non-motor symptoms Questionnaire; OL: open-label; Ona: OnabotulinumtoxinA; P: prospective; PC: placebo-controlled; QoL: quality of life; R: retrospective; Ra: randomized; RCT: randomized controlled trial; Rima: Rimabotulinumtoxin-A; RS: rating scale; SB: single-blind; SC: single-center; VAS: visual analogue scale.

**Table 2:** BoNT comparative studies in hemifacial spasm.

	Author	Design	Clinical Evaluation	*n*°	Mean Age (years)	Disease Duration (years)	Comparison	Conversion Ratio	Mean Dosage (UI)	Improvement	Mean Effect Duration	Comments
1	Marion (1995) [55]	P, OL, DF	n/a	37	54.6	8.16	Abo vs. Ona	3:1	85 (Abo)	n/a	n/a	Similar effects
									32 (Ona)			
2	Sampaio (1996) [56]	P, Ra, SC, SB	BRS	49	58.2 (Abo)	6.13 (Abo)	Abo vs. Ona	1:4	n/a	n/a	13.9 weeks (Abo)	Similar effects
					63.2 (Ona)	3.99 (Ona)					13.4 weeks (Ona)	
3	Bihari (2005) [57]	P, SA, CO	SA	9	53.5	n/a	Abo vs. Ona	5:1	16 (Ona)	77%	65.1 days (Ona)	more effective Ona
									78 (Abo)	60%	41.8 days (Abo)	
4	Rieder (2007) [58]	P, Ra, CO, DB	SA	18	60.23	8.8	Lan vs. Ona	1:1	n/a	n/a	72 days (Lan)	No differences
											71 days (Ona)	
5	Dressler (2009) [59]	CO, R/P	SA	11	61.1	6.8	Ona vs. Inco	1:1	43.3	n/a	n/a	No differences
6	Bentivoglio (2009) [4]	R, SC	SA	108	54.1	7.9	Ona vs. Abo	n/a	11.2 (Ona)	94%	105.4 days (Ona)	No differences
									46.5 (Abo)		85.4 days (Abo)	
7	Quagliato (2010) [60]	P, Ra, DB	HFSES, SF-36	17	59.8	9.1	Ona vs. Lan	1:1	35	n/a	12.8 weeks	No differences
8	Kollewe (2010) [47]	P	GCI	53	n/a	6.0	Ona vs. Abo	1:2,56	22	2.6	12.1 weeks	No differences
9	Wu (2011) [61]	P	Cohen’s scale	131	45.8 (Lan)	n/a	Lan vs. Ona	1:1	n/a	97% (Lan)	16.2 weeks (Lan)	No differences
					45.3 (Ona)					94% (Ona)	16.5 weeks (Ona)	
10	Bentivoglio (2012) [29]	R, SC, DF	SA	10	51.6	12.3	Ona vs. Abo	1:3—1:5	n/a	n/a	n/a	No differences
11	Bladen (2020) [62]	P, SB, M	SA	12	n/a	n/a	Ona vs. Inco	1:1	n/a	84% (Inco)	12 weeks (Inco)	More effective Inco
										72% (Ona)	11 weeks (Ona)	
12	Ozer (2021) [63]	R, SC	VAS	16	53.2	11	Ona vs. Abo	1:4,95	n/a	n/a	n/a	No differences

Abo: AbobotulinumtoxinA; BRS: blepharospasm rating scale; CO: crossover; DB: double-blind; DF: dose-finding; GCI: Global clinical improvement; HFSES: Hemifacial Spasm Evaluation Scale; JRS: Inco: IncobotulinumtoxinA; Jankovic Rating Scale; Lan: LanbotulinumtoxinA; M: multicenter; OL: open-label; Ona: OnabotulinumtoxinA; P: prospective; R: retrospective; Ra: randomized; SA: subjective assessment; SB: single-blind; SC: single-center; SF-36: 36-Item Short-Form Health Survey questionnaire; VAS: visual analogue scale.
